# Anterior Segment Characteristics and Quality of Life of Patients with Central Serous Chorioretinopathy

**DOI:** 10.3390/jcm14061812

**Published:** 2025-03-07

**Authors:** Hadas Ben-Eli, Tal Asher, Rivkah Lender, Devora Mirsky, Riad Abu-Shkara, Mahmud Hamuda, Nadin Aslee, Hadeel Marei, Reut Flug, Renana Eitan, Samer Khateb

**Affiliations:** 1 Department of Ophthalmology, Hadassah-Hebrew University Medical Center, Jerusalem 9112001, Israel; 2 Department of Optometry and Vision Science, Jerusalem Multidisciplinary College, 37 Neviim St., Jerusalem 9101001, Israel; 3 Geha Mental Health Center, 1 Helsinki St., Petah Tikva 4910002, Israel; 4 The Jerusalem Mental Health Center, The Hebrew University, Jerusalem 9112102, Israel

**Keywords:** central serous chorioretinopathy, diabetic retinopathy, anterior segment, quality of life, anxiety

## Abstract

**Background**: This study aimed to compare the anterior segment characteristics of patients with central serous chorioretinopathy (CSCR) to those with diabetic retinopathy (DR) and healthy controls. Additionally, it explored the possible associations between quality of life and anxiety with CSCR. **Methods**: A single-center, cross-sectional study involving patients aged 23–61 years diagnosed with CSCR or DR, and healthy patients. Comprehensive ophthalmic examinations included best-corrected visual acuity (BCVA, LogMAR), objective and subjective refraction, and anterior and posterior segments optical coherence tomography (OCT) imaging. Participants completed the Quality-of-Life Enjoyment and Satisfaction Questionnaire (Q-LES-Q) and the Beck Anxiety Inventory (BAI). Statistical analysis included Kruskal–Wallis, Tukey post-hoc, Chi-square, and Spearman correlation tests to compare the three groups. **Results**: A total of 53 patients were recruited (16 CSCR, 8 DR, 29 controls; 52.8% males), with an additional 16 CSCR patients completed only the questionnaires. CSCR and DR patients were the same age as the controls (43.8 ± 9.0, 42.7 ± 9.9, 37.06 ± 13.61 years, respectively, *p* = 0.19). CSCR and DR patients had similar BCVA, lower than controls (0.19 ± 0.30, 0.15 ± 0.13, 0.01 ± 0.02 LogMAR, respectively, *p* < 0.01). CSCR patients exhibited more hyperopic refraction compared to healthy controls (*p* < 0.01) and reported significantly lower life enjoyment and satisfaction than DR and healthy individuals (51.56 ± 9.17, 53.75 ± 7.81, 60.03 ± 7.32, respectively, *p* < 0.01). No significant correlations were found between anxiety levels and pupil size, anterior chamber depth (ACD), amplitude of accommodation (AA), and intraocular pressure (IOP) among study groups (*p* > 0.05). **Conclusions**: CSCR patients demonstrated lower life enjoyment and satisfaction, reduced BCVA, and hyperopic refraction compared to healthy patients. They also tended to have higher stress and anxiety levels. Both CSCR and DR patients shared similar anterior segment characteristics.

## 1. Introduction

Central serous chorioretinopathy (CSCR) is a retinal disorder characterized by localized serous detachment of the neurosensory retina in the macula, leading to macular edema [1]. CSCR predominantly manifests unilaterally in young to middle-aged individuals, with its highest prevalence observed around the age of 45 years [1]. Notably, CSCR exhibits a male predilection [2]. The acute phase of the disease most commonly undergoes spontaneous resolution within a span of four to six months [3].

Patients affected with CSCR frequently present with visual disturbances, including metamorphopsia, micropsia, dyschromatopsia, and a decline in visual acuity and contrast sensitivity [2,4]. Optical coherence tomography (OCT) scans demonstrate subretinal fluid accumulation and retinal pigment epithelium (RPE) detachments, while fluorescein angiography (FA) highlights the presence of various patterns of leakage [5]. Numerous risk factors have been postulated as potential contributors to CSCR, including systemic steroids, pregnancy, antibiotics, alcohol consumption, untreated hypertension, allergic respiratory disease [6], heightened stress levels [1], anxiety, and depression [7].

Type A personality disorder is not recognized as a formal psychiatric diagnosis in the Diagnostic and Statistical Manual of Mental Disorders (DSM-5) or other major diagnostic manuals. However, the term “Type A personality” has been used informally to describe a set of behavioral traits and characteristics that are associated with a high level of competitiveness, ambition, impatience, hostility, and a sense of urgency. Of particular significance to the current study, individuals exhibiting a Type A personality demonstrate increased susceptibility to CSCR [8,9,10]. It is worth noting that Type A personality has been reported to be associated with an increased risk of various health conditions, including cardiovascular diseases [9], hypertension [10], CSCR, and psychological factors such as anxiety and depression [1,7]. It has been previously demonstrated that in both resting state and response to challenging situations, Type A behavior patterns were associated with more enhanced sympathetic reactivity and suppressed parasympathetic activity than Type B, which is known for being more relaxed, laid-back, and less prone to stress and urgency [1,8,11,12]. Moreover, Type A subjects showed a significant reduction in muscle tension in both resting and aroused states [13]. This may affect visual acuity (VA), pupil diameter, intraocular pressure (IOP), and activation of accommodation [8], and cause decreased aqueous humor production [14].

A preliminary study found that patients with Type A personalities exhibit greater lag of accommodation in the Fused Cross Cylinder (FCC) test [15]. Moreover, biometric parameters were previously reported to be associated with other retinopathies, such as in Type 2 diabetes mellitus [16,17]; however, no comparison has been carried out between CSCR and other retinopathy patients. In the current study, we aimed to compare additional anterior segment and refractive parameters among CSCR patients vs. healthy controls and diabetic retinopathy (DR) patients, as well as to examine the possible association between CSCR, degree of life satisfaction, and anxiety characteristics. The comparison with the DR group allows us to further elucidate which characteristics are unique to CSCR and which are shared by other retinopathies.

## 2. Methods

### 2.1. Study Design and Patient Recruitment

A cross-sectional, single-center study was conducted as per the tenets of the Declaration of Helsinki and was approved by the institutional Helsinki committee of the Hadassah-Hebrew University Medical Center (Study #: HMO-19-230). All patients provided informed consent prior to their participation. A total of 69 patients, aged between 23–61 years old, were recruited from the retina clinics, the Department of Ophthalmology, Hadassah Medical Center, between August 2021 and March 2023. Participants were divided into three groups: CSCR, DR patients, and healthy patients.

Inclusion criteria were as follows: no previous ocular surgeries, laser treatment, or intraocular injections, with BCVA ≤ 0.3 LogMAR for distance, J1+ on the Jaeger near chart, and normal binocular vision, assessed by the Paul Harris Randot Test with a threshold of 40 Seconds of Arc (SOA). The CSCR group included patients who were diagnosed with active CSCR in at least one eye based on anamnesis, fundoscopy, and posterior segment OCT scan demonstrating subretinal fluid (SRF), and the DR group included patients with established mild non-proliferative diabetic retinopathy (NPDR) without macular edema for at least one year prior to recruitment [18].

Pregnant women, uncontrolled chronic diseases such as diabetes, epilepsy, or hyperthyroidism, and patients who were unable to understand the consent form or were unwilling to sign it were excluded.

Due to the substantial interocular correlation documented in the literature [19], only the right eyes of DR and healthy participants were included in the data analysis. However, all eyes with active CSCR were included in the analysis.

### 2.2. Ocular Examination

A comprehensive set of tests was conducted by a retina specialist, including biomicroscopy and dilated fundoscopy. The tests included objective autorefraction (Wave analyzer WAM700, Essilor Ltd., Dallas, TX, USA), subjective refraction, near (Jaeger) and distance (LogMAR) BCVA, stereopsis evaluation using the Paul Harris Randot Test (SOA), measurement of accommodative lag using the FCC test (Diopters (D)), and quantification of the amplitude of accommodation using the push-away method (D).

### 2.3. Optical Coherence Tomography (OCT) Imaging

Anterior segment (AS) OCT scans were obtained using the CASIA2 AS-OCT (Tomey Corporation, Nagoya, Japan). These scans provided the ocular anterior segment parameters such as pupil size under mesopic conditions (mm), anterior chamber depth (ACD), and angle size, represented by the Trabecular–Iris Angle (TIA) at 500 µm both nasally and temporally. Horizontal cross-sections of posterior segment OCT imaging were obtained using Spectral Domain-OCT (Heidelberg Engineering, Heidelberg, Germany).

### 2.4. Q-LES-Q and Beck Anxiety Inventory (BAI)

Each participant was asked to fill out a Quality-of-Life Enjoyment and Satisfaction Questionnaire (Q-LES-Q) [20] and a Beck Anxiety Inventory (BAI) [21]. The Q-LES questionnaire, designed to assess life satisfaction and enjoyment, is comprised of 14 questions, rated on a scale of 1 to 5, with the highest value being indicative of the greatest satisfaction. The final scores range from 14 to 70, with higher scores denoting greater life satisfaction [20]. The Beck Anxiety Inventory, designed to gauge anxiety traits, includes 21 items and is rated on a four-point Likert scale (0–3). Final scores range from 0 to 63 and are divided into four categories: minimal (0 to 7), mild (8 to 15), moderate (16 to 25), and severe anxiety (26 to 63) [21]. The five most representative questions in the BAI that cover the common domains of anxiety symptoms are numbness or tingling, fear of losing control, difficulty breathing, worry about the future, and fear of dying.

### 2.5. Statistical Analysis

Descriptive statistical methodologies were employed, with the Kolmogorov–Smirnov test used to evaluate the normality distribution of study parameters. Differential variations across the study groups were assessed using the non-parametric Kruskal–Wallis and Chi-square tests. In instances where statistically significant differences were discerned among the three study groups, the Tukey HSD post-hoc test was subsequently employed to pinpoint disparities between any two study groups. To probe interrelationships between variables, the Spearman coefficient was employed for correlation analysis. A statistically significant outcome was ascribed to instances where the *p*-value was α < 0.05 for two-sided tests. The analysis was performed using SPSS software (IBM SPSS Statistics, Version 27.0, IBM Corp: Armonk, NY, USA).

## 3. Results

### 3.1. Ocular Characteristics

**Figure 1:**
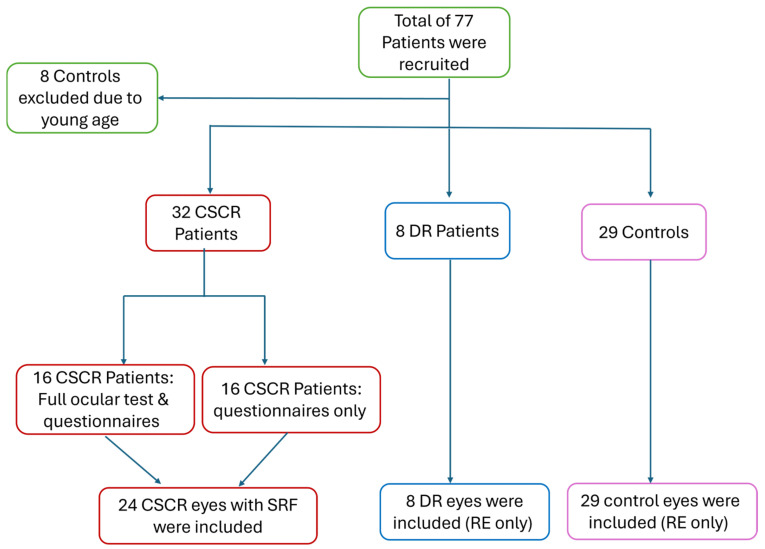
**Study flowchart demonstrating patient recruitment into each of the study groups.** RE = Right eye; RSF = subretinal fluid.

A total of 69 patients were recruited into the study, comprising 32 CSCR patients (of which 16 participated in the questionnaire only (14 males, 87.5%)), 8 DR patients (4 males, 50%), and 29 healthy participants (10 males, 34.5%). A high correlation was observed between both eyes for the tested parameters across all study participants (r ranging from 0.49 to 0.99; *p* < 0.01). Therefore, the right eyes of DR (N = 8 eyes) and healthy patients (N = 29 eyes) were included, while all eyes presenting with SRF in the CSCR group were included (N = 24 eyes, including both eyes of 8 patients; Figure 1 and Table 1). All participants were aged 23–61 years old. There was no difference in mean age between study groups (43.8 ± 9.0, 42.7 ± 9.9, 37.06 ± 13.61 years; respectively, *p* = 0.19). As expected, the LogMAR visual acuity of CSCR patients was similar to DR but lower than the control group (0.19 ± 0.30, 0.15 ± 0.13, 0.01 ± 0.02, respectively; *p* < 0.01; Table 1), highlighting the effect of the background retinopathy on the BCVA. Additionally, refraction of the CSCR patients demonstrated a more hyperopic spherical equivalent compared to DR and control groups (0.33 ± 1.14, −1.78 ± 2.97, −1.68 ± 2.68, respectively; *p* < 0.01; Table 1).

**Figure 2:**
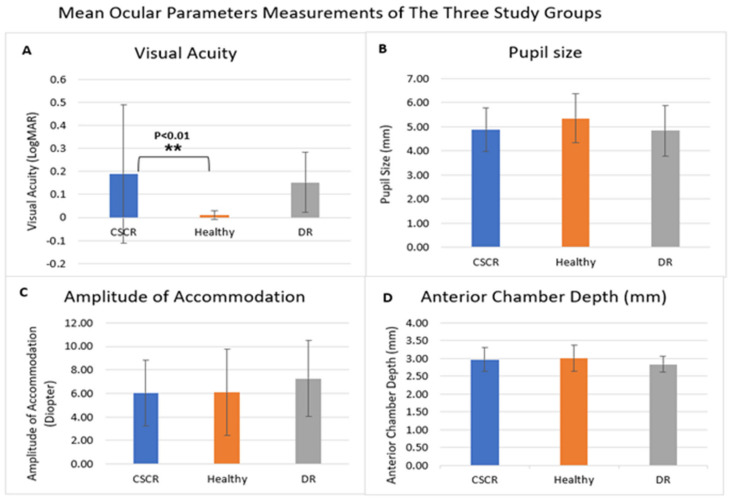
**Comparison of the mean values of various ocular parameters measured in the study groups.** CSCR patients had lower VA (**A**) compared to healthy controls. No significant differences were found between the study groups in terms of pupil size (**B**), amplitude of accommodation (AA) (**C**), and anterior chamber depth (ACD) (**D**). ACD = Anterior chamber depth.

**Figure 3:**
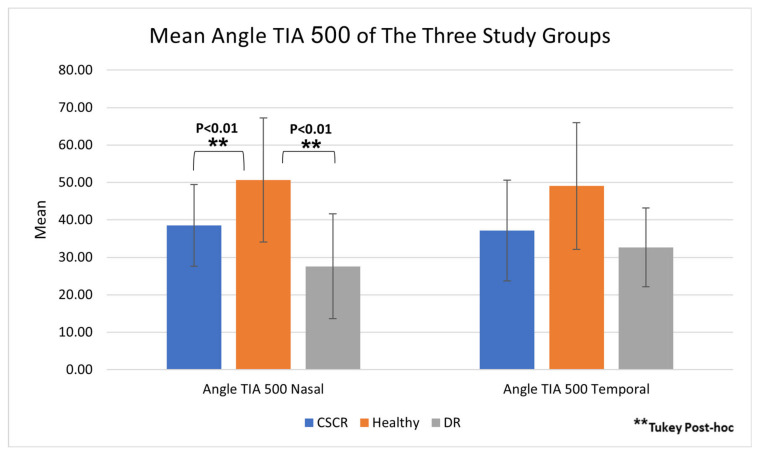
**Comparison of the mean angle TIA 500 for the 3 study groups.** CSCR and DR patients had statistically significant narrower nasal TIA than healthy controls. TIA = Trabecular–Iris angle.

Comparison of anterior segment parameters demonstrated no statistically significant difference in pupil size measured by AS-OCT for the CSCR and DR patients compared with healthy patients (4.88 ± 0.89, 4.84 ± 1.04, 5.35 ± 1.02, respectively; *p* = 0.16; Table 2). In addition, no statistically significant difference was found in anterior chamber depth (ACD) between the study groups (2.97 ± 0.34, 2.83 ± 0.22, 3.01 ± 0.36, respectively; *p* = 0.31; Table 2). Of note, statistically significant differences were identified between study groups in TIA: CSCR and DR patients had narrower nasal TIA than healthy controls (38.52 ± 10.93, 27.61 ± 14.02, 50.65 ± 16.57, respectively; *p* < 0.01; Table 2 and Figure 2), yet no difference was noticed between CSCR and DR groups in nasal TIA (*p* = 0.15, Figure 3). In addition, the temporal TIA was similar between study groups (*p* = 0.30). The IOP and Amplitude of Accommodation were similar among the three study groups (*p* = 0.96 and *p* = 0.70, respectively).

Comparison of anterior segment parameters between the two eyes of CSCR patients in whom only one eye was affected with SRF demonstrated no difference in ACD (*p* = 0.95) and pupil size (*p* = 0.83).

### 3.2. Q-LES-Q and Beck Anxiety Inventory Analyses

An additional 16 CSCR patients (14 males, 87.5%) who were previously recruited for a Q-LES-Q and Beck Anxiety Inventory study that did not include the anterior segment workup were included in the final analysis. Hence, a total of 32 CSCR subjects, 8 DR subjects, and 29 controls were included in the life satisfaction and anxiety traits score analyses.

A significantly lower Q-LES-Q score, indicating decreased life satisfaction, was found among CSCR patients compared to healthy controls (51.56 ± 9.17, 60.03 ± 7.32, respectively; *p* < 0.001; Table 3). Of note, no difference was found between CSCR and DR groups (*p* = 0.76), indicating similar Q-LES-Q results for patients suffering from two different retinopathies.

**Figure 4:**
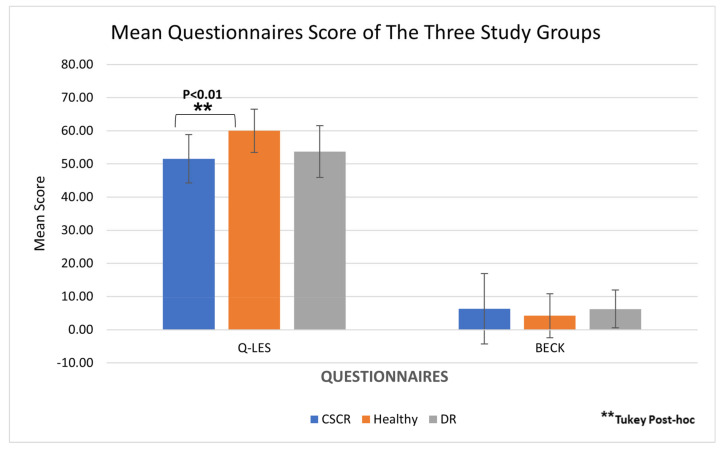
**Mean questionnaire scores of the three study groups.** The CSCR group demonstrated lower life satisfaction compared to healthy controls and showed a tendency towards a higher prevalence of stress and anxiety.

The Beck Anxiety Inventory demonstrated no difference between the three study groups both in terms of personal anxiety patterns (*p* = 0.19) and sub-classification to severity of anxiety patterns (*p* = 0.54). Furthermore, no differences were found even when the five most representative questions were analyzed separately (*p* = 0.33) (Table 3 and Figure 4).

As expected, a statistically significant inverse correlation was found between the Q-LES-Q and Beck Anxiety Inventory (r = −0.58, *p* < 0.01): the greater the stress and anxiety levels, the lower the satisfaction with life. Weak and non-significant correlations were found between Q-LES-Q and the Beck Anxiety Inventory and the measured ocular parameters (−0.02 to −0.25; *p* > 0.05).

No difference was found in the FCC results (*p* = 0.12) between the study groups, indicating that the preliminary observation regarding the greater accommodative lag among patients with Type A behavior patterns is not replicated in CSCR patients despite former evidence of high prevalence of this personality type in this patient group.

## 4. Discussion

This study aimed to compare the anterior segment characteristics of patients with CSCR to those with DR and healthy controls. Additionally, the study investigated potential associations between quality of life, anxiety, and retinopathy.

Previous research has explored the interplay of ocular, systemic, and psychological factors in CSCR, contributing to a better understanding of its pathophysiology. For example, a preliminary study by Ben-Eli et al. identified significant gender-related differences associated with personality type and fused cross-cylinder (FCC) measures. Specifically, females exhibiting a Type A behavior pattern showed a significantly higher accommodative lag in FCC (*p* < 0.04) [15]. As CSCR has also been linked to Type A personality traits [22], this study sought to evaluate anterior segment parameters, as measured by anterior segment optical coherence tomography (AS-OCT), in CSCR patients relative to healthy individuals and those with background DR. Furthermore, the study examined associations between ocular structural characteristics, anxiety levels, and life satisfaction, aiming to integrate anatomical and psychological aspects in the characterization of the disease.

Our findings demonstrated that there is no significant difference in the pupillary size, anterior chamber depth, and amplitude of accommodation between both study groups and the control group. Our findings, which were measured accurately using AS-OCT, are in line with a previously reported study by Zhou et al., who showed similar baseline pupillary diameter size between CSCR and control groups. At the same time, CSCR patients demonstrated smaller constriction amplitude and a higher re-dilation ratio compared to the control group when performing near-tasking [23]. This was attributed to extra sympathetic activation and attenuated parasympathetic response among CSCR patients. Of note, being a risk factor for CSCR, anxiety increases norepinephrine blood levels due to sympathetic system activation [24]. A recently published study pinpointing risk factors for small pupil size found an association with older age, hyperopic refractive error, previous cataract surgery, ACE inhibitor use, diabetes, and obesity, whereas larger pupils were associated with arterial hypertension, female sex, tricyclic antidepressant use, and tetracyclic antidepressant use [25]. An explanation for this may be related to the fact that the pupillary sphincter muscle can be relaxed by serotonin, noradrenergic, and anticholinergic effects [26]. Since each of our study groups contains different risk factors that affect pupil size in opposite ways, this may mask differences between the different groups. Diabetes and hyperopia cause small pupils, while young age and male gender cause larger pupils.

The absence of significant differences in pupillary size between the study groups may reflect the multifactorial nature of CSCR. While sympathetic activation and parasympathetic attenuation are implicated in pupillary regulation, their roles in CSCR remain unclear. Neurological system effects might be more evident in dynamic pupillary responses rather than static anatomical structures. Previous studies have reported smaller pupil sizes and reduced amplitude of pupillary responses in diabetic patients [27], suggesting that such changes are likely general consequences of retinopathies rather than specific to CSCR. However, this was not observed in the current study population.

An additional finding in this study was mild hyperopic shift identified in CSCR patients compared to controls, while no significant difference was noted for anterior chamber (AC) depth. A similar hyperopic shift was reported in recent research by Gawęcki et al., despite comparable axial lengths between CSCR patients and controls [28]. The data regarding the AC depth is controversial. All study groups demonstrated similar AC depth, although the nasal trabecular–iris angle in the CSCR group was shallower. Previous studies showed shallow AC depth for acute CSCR [29]. The hyperopic shift observed here could also be influenced by age-related cataract development, alteration in lens position, and/or macular edema, as these parameters may be causally linked. No other significant structural abnormalities were identified that could elucidate a specific pathological mechanism in CSCR.

CSCR patients demonstrated lower life satisfaction compared to control subjects while being similar to the DR group as assessed by the Quality-of-Life Enjoyment and Satisfaction Questionnaire (Q-LES-Q). For the Beck Anxiety Inventory, a trend towards higher levels of stress and anxiety was observed as the mean score of the CSCR group was higher than the control group but still not statistically significant. Previous studies have identified severe difficulties in emotional regulation among CSCR patients, including neuroticism [30], emotional instability, introversion [31], and Type A behavior. However, this is the first study to evaluate life satisfaction and anxiety using validated questionnaires [8]. Retrospective studies have noted increased psychological distress in CSCR patients compared to healthy controls, though the causality between distress and CSCR remains unclear [32]. Chronic diseases are well documented to influence depressive symptoms, which may in turn affect life satisfaction [33].

Balkarli et al. found higher Beck Anxiety Inventory scores in CSCR patients with fibromyalgia syndrome (FMS) compared to those without FMS, indicating greater anxiety severity when systemic diseases co-occur with CSCR [34]. The higher prevalence of FMS among CSCR patients of both sexes, along with the uncertain pathophysiology of CSCR, underscores the importance of evaluating these patients for coexisting systemic conditions. Addressing modifiable risk factors may play a critical role in reducing the recurrence of CSCR episodes [34].

This study has several limitations, including a small sample size, which was accounted for using appropriate statistical analyses. Strengths of the study include its cross-sectional design and the use of two control groups (healthy individuals and diabetic retinopathy patients), enabling differentiation between findings specific to CSCR and those associated with retinopathy more broadly. Furthermore, integrating structural and psychological assessments offered a comprehensive perspective on the risk factors associated with CSCR development and progression.

## 5. Conclusions

This study explored the relationship between anterior segment features measured by AS-OCT and the anxiety and life satisfaction traits of CSCR patients. Decreased life satisfaction, reduced visual acuity, hyperopic refractive shifts, and shallow nasal trabecular–iris angle were identified in CSCR patients in the CSCR group compared to healthy controls. Although no significant differences in pupillary size and amplitude of accommodation were observed, CSCR patients exhibited a trend towards higher levels of stress and anxiety. These findings highlight the need for large-scale, population-based studies to further investigate systemic and ocular risk factors for CSCR.

## Figures and Tables

**Table 1:** Basic characteristics of the study population.

	CSCR	DR	Controls	*p*-Value
	N = 24 Eyes	N = 8 Eyes	N = 29 Eyes	
**Mean Age ± SD (years)**	43.8 ± 9.06	42.7 ± 9.96	37.06 ± 13.61	0.19 ^a^
**Age range (years)**	23–55	24–55	23–61	N/A
**Male gender n (%)**	14 (87.5%)	4 (50%)	10 (34.48%)	**<0.01 ^b^**
**BCVA (LogMAR) Mean ± SD**	0.19 ± 0.30	0.15 ± 0.13	0.01 ± 0.02	**<0.01 ^a^**Post Hoc: DR vs. Healthy 0.17 ^c^ DR vs. CSCR 0.87 ^c^ CSCR vs. Healthy **<0.01 ^c^**
**SPH (Diopter) Mean ± SD**	0.67 ± 1.08	−0.13 ± 2.85	−1.44 ± 2.76	**<0.01**^a^ Post Hoc: DR vs. Healthy 0.32 ^c^ DR vs. CSCR 0.66 ^c^ CSCR vs. Healthy **<0.01**^c^
**Cyl (Diopter) Mean ± SD**	−0.77 ± 0.43	−1.31 ± 1.15	−0.49 ± 0.62	**<0.01**^a^ Post Hoc: DR vs. Healthy **<0.01 ^c^** DR vs. CSCR 0.11 ^c^ CSCR vs. Healthy 0.26 ^c^
**Spherical Equivalent (Diopter) Mean ± SD**	0.3 ± 1.14	−1.78 ± 2.97	−1.68 ± 2.68	**<0.01 ^a^** Post Hoc: DR vs. Healthy 0.99 ^c^ DR vs. CSCR 0.06 ^c^ CSCR vs. Healthy **<0.01 ^c^**

SD = Standard Deviation; VA = Visual Acuity; dec = decimal; SPH = sphere; Cyl = Cylinder. ^a^ Kruskal–Wallis Test; ^b^ Chi-square test; ^c^ Tukey HSD Post-hoc Test. The Bold values indicate the statistical significance of *p* value < 0.05.

**Table 2:** Comparison of ocular parameters between study groups.

	CSCR	DR	Controls	*p*-Value
	N = 24 Eyes	N = 8 Eyes	N = 29 Eyes	
**CASIA Pupil Size (mm)** **Mean ± SD**	4.88 ± 0.89	4.84 ± 1.04	5.35 ± 1.02	0.16 ^a^
**ACC (Diopter)** **Mean ± SD**	6.04 ± 2.78	7.27 ± 3.68	6.09 ± 3.24	0.70 ^a^
**ACD (mm)** **Mean ± SD**	2.97 ± 0.34	2.83 ± 0.22	3.01 ± 0.36	0.31 ^a^
**IOP (mmHg)** **Mean ± SD**	13.16 ± 2.16	13.25 ± 2.55	14.16 ± 2.98	0.51 ^a^
**Nasal Angle TIA 500 ** **Mean ± SD**	38.52 ± 10.93	27.61 ± 14.02	50.65 ± 16.57	**0.02 ^a^** Post Hoc: DR vs. Healthy **<0.01 ^b^** DR vs. CSCR 0.15 ^b^ CSCR vs. Healthy **<0.01 ^b^**
**Temporal Angle TIA 500** **Mean ± SD**	37.16 ± 13.44	32.65 ± 10.53	49.07 ± 16.92	0.30 ^a^

SD = Standard Deviation; FCC = fused cross cylinder; ACD = anterior chamber depth; IOP = intraocular pressure; TIA = Trabecular–Iris Angle. ^a^ Kruskal–Wallis Test; ^b^ Tukey HSD Post-hoc Test. The Bold values indicate the statistical significance of *p* value < 0.05.

**Table 3:** Comparison of subjective questionnaire results across study groups.

	CSCR N = 32 Patients	DR N = 8 Patients	Controls N = 29 Patients	*p*-Value
**Q-LES questionnaire** **Mean ± SD**	51.56 ± 9.17	53.75 ± 7.81	60.03 ± 7.32	**<0.01 ^a^** Post Hoc: DR vs. Healthy 0.14 **^b^** DR vs. CSCR 0.78 **^b^** CSCR vs. Healthy **<0.01 ^b^**
**BECK questionnaire** **Mean ± SD**	6.31 ± 8.50	6.25 ± 5.70	4.21 ± 6.99	0.19 ^a^
**BECK questionnaire (4, 5, 9, 14, 17)** **Mean ± SD**	2.12 ± 3.15	1.25 ± 2.05	1.10 ± 2.00	0.54 ^a^

SD = Standard Deviation. ^a^ Kruskal–Wallis Test; ^b^ Tukey HSD Post-hoc Test. The Bold values indicate the statistical significance of *p* value < 0.05.

## Data Availability

The datasets generated and/or analyzed during the current study are available from the corresponding author upon reasonable request. Additional details regarding the research materials can also be provided upon request. All data are stored following ethical guidelines and privacy regulations to ensure the confidentiality and integrity of participant information.

## Abbreviations

VA	Visual Acuity
CSCR	Central Serous Chorioretinopathy
DR	Diabetic Retinopathy
OCT	Optical Coherence Tomography
AS-OCT	Anterior Segment Optical Coherence Tomography
Q-LES-Q	Quality-of-Life Enjoyment and Satisfaction Questionnaire
BAI	Beck Anxiety Inventory
